# Labor/leisure decisions in their natural context: The case of the smartphone

**DOI:** 10.3758/s13423-020-01844-2

**Published:** 2020-11-20

**Authors:** Jonas Dora, Madelon van Hooff, Sabine Geurts, Michiel Kompier, Erik Bijleveld

**Affiliations:** Behavioural Science Institute, Radboud University, P.O. Box 9104, 6500 HE Nijmegen, The Netherlands

**Keywords:** Labor/leisure decision, Smartphone, Notification, Distraction, Ecological validity

## Abstract

In this research, we attempt to understand a common real-life labor/leisure decision, i.e., to perform cognitive work or to interact with one’s smartphone. In an ecologically valid experiment, participants (*N* = 112) could freely switch back and forth between doing a 2-back task and interacting with their own smartphone. We manipulated the value of the 2-back task (by varying the value of monetary rewards; within-subjects) and of the smartphone (by switching on and off airplane mode; within-subjects) while we recorded incoming notifications, such as text messages. Our study produced three main findings: (1) the current value of the smartphone did not increase our statistical model’s ability to predict switches from labor to leisure when the current task value was also taken into account; (2) however, participants reacted strongly to naturally incoming notifications, which were the strongest predictor of labor-to-leisure switches; (3) there was no evidence that taking into account individual differences (in the value assigned to labor and leisure) improved the model’s ability to predict labor-leisure switches. In sum, using a situated approach to studying labor/leisure decisions, our findings highlight the importance of high task motivation, as well as the temporary distractive potential of smartphone notifications, when people face the challenge of staying focused on their productive tasks.

## Introduction

In everyday life, people are often confronted with *labor/leisure decisions*. For example, students may be faced with the decision whether to continue studying (labor) versus to idle on their smartphones (leisure); office workers may be faced with the decision to continue answering client emails (labor) versus to plan their next vacation (leisure); and even in their free time, people may have to decide whether to go to the gym (labor) versus to stay at home and watch Netflix (leisure). Against the background of current societal changes, for example, work is becoming more mentally demanding (Goldman & Scardamalia, 2013) and smartphones offer instant entertainment (Oulasvirta, Rattenbury, Ma, & Raita, 2012) – it is important to understand how people make these labor/leisure decisions. In this study, we aimed to understand one very ubiquitous labor/leisure decision, namely the decision to pick up one’s smartphone while one is doing cognitive work.

Labor/leisure decisions are defined as decisions between working hard to obtain some reward (mental labor) and carrying out an undemanding activity that provides relief (mental leisure; Kool & Botvinick, 2014; for a discussion of how the labor/leisure model relates to other models of motivation, see Inzlicht, Schmeichel, & Macrae, 2014). Previous research on labor/leisure decisions yielded several important insights: First, research suggested that labor/leisure decisions can be best understood by analyzing the costs and benefits of both labor and leisure options. For example, people’s labor/leisure decisions depend on the current value of labor (e.g., the monetary rewards that are tied to working; Kool & Botvinick, 2014) and the current value of leisure (e.g., having available a pleasant alternative to labor; Rom, Katzir, Diel, & Hofmann, 2019). Second, the feeling of fatigue seems to be a key driver of the decision to disengage from a demanding task, in that people are more likely to switch from labor to leisure when they feel more fatigued (Dora, van Hooff, Geurts, Kompier, & Bijleveld, 2019; Dora, van Hooff, Geurts, Kompier, & Bijleveld, 2020; see Hockey, 2011). Third, labor/leisure decisions are idiosyncratic, in that individual differences matter. For example, people in higher need for cognition (i.e., people who value labor more) tend to choose labor even if the reward for labor is lower (Westbrook, Kester, & Braver, 2013). Moreover, people differ strongly in the relative value they assign to different leisure alternatives to the same labor task (Dora et al., 2019). Together, consistent with recent theories (Inzlicht et al., 2014; Kurzban et al., 2013; Shenhav et al., 2017), these studies suggest that labor/leisure decisions are determined by a cost-benefit analysis process – that does not necessarily take place within conscious awareness (Kurzban et al., 2013) – in which people weigh the value of the current activity (e.g., labor) against the value of the next-best alternative (e.g., leisure).

Although these initial studies are important – after all, they yielded mechanistic insight – all have attempted to understand labor/leisure decisions in artificial, highly controlled contexts. As a result, these previous studies have helped to understand how a small number of variables affect labor/leisure decisions when they are made in the laboratory. However, it is currently less clear how people make labor/leisure decisions in a more ecologically valid setting (Lin, Werner, & Inzlicht, 2020).

Here we present a laboratory experiment in which we prioritized ecological validity in all design decisions. We study the context of one specific labor/leisure decision, namely the decision between doing a productive-but-demanding cognitive task (labor) and interacting with one’s own smartphone (leisure). In doing so, we add to the literature on labor/leisure tradeoffs in three novel ways. First, previous experiments either constrained when and how often people could switch back and forth between labor and leisure, or gave people the choice between two artificial tasks that differed only in mental demands required to perform them. In this experiment, as in real life, participants can freely switch back and forth between labor and leisure tasks. Second, as in previous work, we manipulate two parameters that often vary in real life: the current value of the task, and the current value of the leisure (smartphone) alternative. However, going beyond previous work, we also take into account that, in real life, the value of smartphone interactions changes with incoming notifications, such as text messages, and that there are individual differences in how much people value smartphone interactions. Third, prior work aimed to isolate the role of individual predictors on labor/leisure decisions. However, real life is more complex. So, rather than explicitly testing the effect of individual variables on the decision to switch, we compared models of varying complexity that provide broader explanations of switches from labor to leisure. With this procedure, we aimed to reveal the *combination* of variables that best explains when people switch from labor to leisure. In summary, with our design choices we strived to improve ecological validity; our study can thus help to advance understanding of labor/leisure decisions in their natural context (Clancey, 1997; Hutchins, 1995; Markman & Dietrich, 2000).

We designed a choice task based on previous work on labor/leisure decisions (Algermissen et al., 2019; Dora et al., 2019; Kool & Botvinick, 2014). In our task, participants can freely switch back and forth between a 2-back memory task (labor) and interacting with their own smartphone (leisure). The 2-back task displays letters to the screen in quick succession and requires participants to react whenever the letter currently displayed is the same as the one before the previous one. As such, it is a mentally demanding and highly monotonous task. While perceptions on smartphone use as a leisure task may differ between people, the fact that it is a relatively undemanding activity paired with a highly demanding cognitive task should ensure that it is uniformly perceived as a leisure task during the experiment. We manipulated the value of the 2-back task by varying the monetary reward associated with performance. We manipulated the value of interacting with the smartphone by activating versus de-activating the *airplane mode* functionality on participants’ smartphones. Activating airplane mode (which prevents access to mobile data and Wi-Fi^1^) should reduce the value of the smartphone, as smartphone users rated a set of smartphone applications that require internet access as significantly more rewarding than a set of applications that do not require internet access (Johannes, Dora, & Rusz, 2019). During the experiment, we measured the number of incoming notifications to participants’ smartphones. Additionally, we measured participants’ trait valuation of mental labor and smartphone-related mental leisure.

## Method

### Preregistration and data availability

We preregistered design, sample size, and statistical analyses. Our preregistration, experimental materials, data, and analysis scripts are available on the Open Science Framework project of this article (https://osf.io/s2wy5/).

### Sample size rationale

We did not perform a power analysis as we did not aim to test any directional hypotheses. Instead, we decided to recruit and run participants either until we would ran out of money (120 participants) or until 1 July 2019. By that date, we had collected data from 112 participants.

### Participants, procedure, and design

One hundred and twelve university students (*M*_*age*_ = 22.10 years; 74 females) participated in exchange for either €5 or partial course credit and an extra cash payment of €0.008 per 2-back trial in high task-value blocks and €0.002 per 2-back trial in low task-value blocks. Participants had to be between 18 and 30 years of age, and had to own a smartphone. Upon the participant’s arrival, the experimenter ensured that the participant brought his/her smartphone, and that the smartphone was sufficiently charged. After obtaining informed consent, together with the participant, the experimenter turned up the sound volume of the smartphone and turned on push notifications for all installed applications. The experimenter also made sure that participants were familiar with the airplane mode functionality and knew how to turn it on and off.

Next, the participant was seated in a cubicle, and the smartphone was connected to a Button Box via an auxiliary cable (*note:* from the smartphone’s perspective, this is as if headphones are connected). The experimenter then sent a text message to the participant’s smartphone to check whether the Button Box correctly recorded the sound signal from the incoming notification. In turn, participants reported demographics (age and gender), received task instructions, and practiced the 2-back task for 20 trials. We instructed participants that they were free to allocate their time between the labor and leisure task, and we mentioned that previous participants had taken multiple smartphone breaks in order to prevent participants from (unnaturally) resisting smartphone use.

Participants then completed eight blocks of the choice task, which is described below. Together, these eight blocks took approximately 40 min to complete. After they were finished, participants were debriefed and received their compensation. The study was approved by the local ethics review board. We employed a 2 × 2 within-subjects design (high task value vs. low task value; high phone value vs. low phone value). We continuously measured incoming notifications, and we continuously recorded participants’ switches from labor to leisure (and vice versa).

### Choice task

The task was scripted with PsychoPy (Peirce, 2007). For labor, we used a visual letter variant of the 2-back task. Participants had to decide whether a letter presented on the screen was a target or a non-target. In case of a target, participants had to press a button on the button box. Targets were defined as trials where the currently presented letter was the same as the letter that was presented before the previous one. The stimuli were presented for 500 ms in the center of the screen, followed by an intertrial interval of 1,500 ms. The target rate was 25%.

One block consisted of 196 2-back trials. In each trial, participants could choose between performing the 2-back task (labor) or interact with their smartphone instead (leisure). Interacting with the smartphone did not prolong the experiment as the trials continued to be counted during leisure. The task value and phone value changed between blocks, with each combination of the task value and phone value manipulations occurring during two blocks. At the beginning of each block, participants learned how much money they could earn per trial (task value); also, they were instructed to either activate or de-activate airplane mode on their phone (phone value). The order of the blocks was randomized. The current combination of task value and phone value was announced at the start of each block and was continuously displayed in the corner of the screen. Participants did not earn money when they interacted with their smartphone. Participants could pause (and unpause) the 2-back task by pressing a corresponding button on the button box. While the task was paused, participants were instructed to do whatever they wanted on their smartphone. Participants could freely switch back and forth between labor and leisure as often and whenever they wanted during each block.

### Questionnaires

#### Need for cognition

We operationalized the trait measure of the value of the labor task by measuring participants’ need for cognition. We used the 18-item Need for Cognition Scale (Cacioppo Petty, & Feng Kao, 1984). The items (e.g., “I would prefer complex to simple problems”; “I find satisfaction in deliberating hard and long for hours”; α = .88) were answered on a 5-point Likert scale from 1 (strongly disagree) to 5 (strongly agree).

#### Need for smartphone use

We operationalized the trait measure of the value of the leisure task by measuring participants’ need for smartphone use. We adapted seven items of the *attitude* subscale of the Media and Technology Usage and Attitude Scale (Rosen, Whaling, Carrier, Cheever, & Rokkum, 2013). This scale measures positive and negative attitudes to, as well as dependence toward, technology. From the original scale, we selected only those items that were specifically related to the smartphone. Due to these seven items having low reliability in our sample (α = .54), we removed the three items that measured negative attitudes. The remaining four items had acceptable internal reliability (α = .70). The items (e.g., “I feel it is important to be able to access my smartphone any time I want”; “I get anxious when I don’t have my smartphone”) were answered on the same 5-point Likert scale.

### Data analysis

We conducted all analyses in R (version 3.6.2; R Core Team, 2020). To test how people make the decision to switch from labor to leisure (i.e., from the 2-back task to the smartphone), we preregistered to run an exhaustive list of Bayesian generalized linear mixed-effects models predicting labor-to-leisure switches from the task value and phone value manipulations. That is, we ran the following five models: (1) an intercept-only model; (2) a model including task value as the sole predictor; (3) a model including phone value as the sole predictor; (4) a model including task value and phone value as predictors; and (5) a model including task value, phone value, and the interaction between the two as predictors.

After fitting these five models, we used the Widely Applicable Information Criterion (WAIC; Watanabe, 2010) of each model to compute Akaike weights. With these weights, we quantified the conditional probability of each model. In other words, we tested which combination of predictors best explains the switch from labor to leisure, while taking increasing model complexity into account (Vehtari, Gelman, & Gabry, 2017). We fitted these models using the *brm* function (*brms* package; version 2.10.0; Bürkner, 2017). In our models, the trial was the unit of analysis. Our categorical, within-subjects predictors were sum-to-zero coded (-1; 1). We used “maximal” random-effects structures in our models (Barr, Levy, Scheepers, & Tily, 2013). Accordingly, our models included a per-participant random intercept to account for the repeated-measures nature of the data. We modeled the within-subjects predictors task value and phone value as fixed effects and as random slopes varying across participants.

For exploratory purposes, we additionally compared models in which we replaced the phone value manipulation with the notifications participants received during the experiment, as well as more complex models involving the trait measures of labor and leisure value. We did not transform the notification data, as generalized mixed-effects models can handle non-normally distributed predictor values (Lo & Andrews, 2015). For all models, we employed the default *brms* priors. For each model, we ran four Markov chain Monte Carlo (MCMC) chains with 4,000 samples. We inspected model fit using the Rhat statistic, the effective sample size, trace plots to make sure that the chains mixed, and posterior predictive checks.

## Results

### Preregistered analyses

On average, during the experiment participants switched 6.13 times from the 2-back task to the smartphone (*SD* = 6.89). They spent roughly 86% of experimental trials engaging in labor, and interacted with their smartphone for the remaining 14% of trials. Participants received an average of 2.28 notifications during the experiment (*SD* = 3.70, min = 0, max = 17). On average, participants reported medium levels of need for cognition (*M* = 3.46, *SD* = 0.54) and need for smartphone use (*M* = 3.08, *SD* = 0.77).

**Fig. 1:**
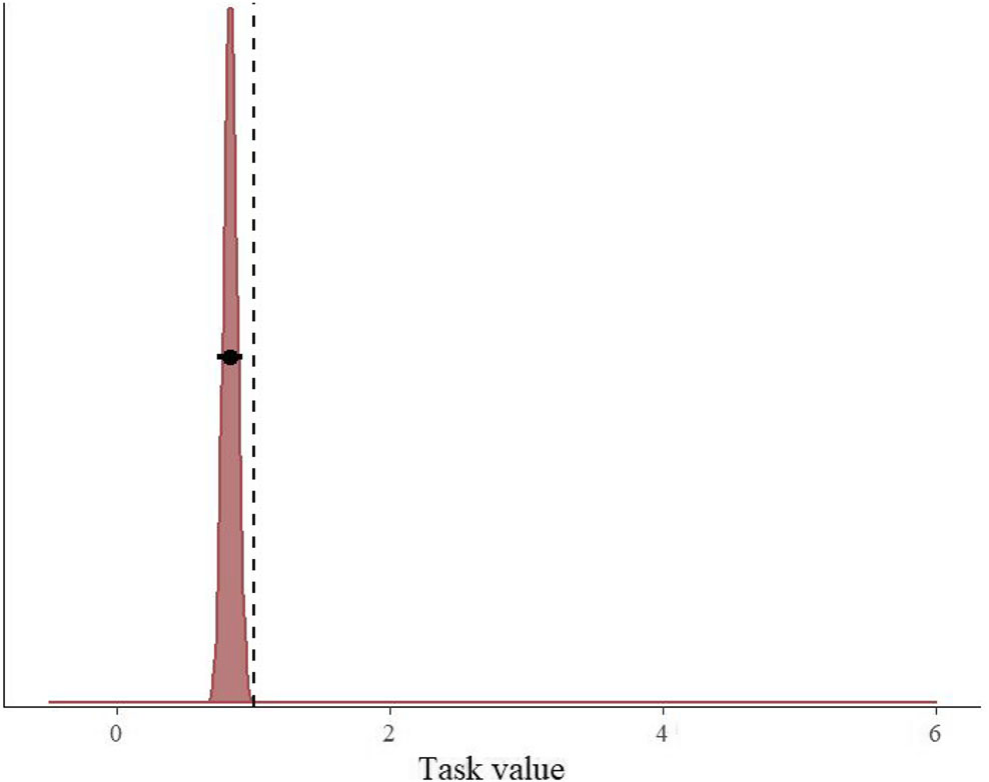
Exponentiated posterior distribution of the parameter (reflecting the odds ratio) for the task value-only model predicting the switch from the 2-back task to the smartphone. The circles and the lines represent the mean of the posterior and the 95% Bayesian credible interval, respectively

Table 1 shows the weights of our five preregistered models predicting the switch from the 2-back task to the smartphone. This analysis revealed that the “winning” model predicting the switch from the 2-back task to the smartphone was the model that included the current task value, but not the current phone value (i.e., whether or not the phone was in airplane mode). This model had the highest estimated probability to explain the labor-to-leisure switch; its weight was almost ten times as large as the weight of the next-best model. In other words, this model’s conditional probability to best explain the switch is almost ten times larger than that of the next model. The posterior distribution of this model can be found in Fig. 1. The model estimates that participants were 1.21 times more likely to switch from the 2-back task to the smartphone if the current task value is low (95% CI = [1.08, 1.36]; 0.83 times more likely if the current task value is high).

**Table 1:** Akaike weights based on Widely Applicable Information Criterion (WAIC) scores of the five task-value/phone-value models predicting the switch from the 2-back task to the smartphone

Model	WAIC	Weight
1 Intercept-only	8,210.9	0.0027
2 Task value	8,199.5	0.8322
3 Phone value	8,215.3	0.0003
4 Task value + phone value	8,204.2	0.0789
5 Task value * phone value	8,204.0	0.0860

### Non-preregistered analyses: Notifications

As the phone value manipulation did not improve the model’s ability to predict the labor-to-leisure switches beyond the task value manipulation, we next explored whether the notifications that participants spontaneously received during the study influenced their decision to switch. For this, we computed two variables. First, we computed the current number of unattended notifications since the last smartphone interaction. Second, we computed a variable that tracked whether or not the participant received a notification in the past ten trials (~20 s). Given that participants could only receive notifications when the airplane mode was deactivated, in these analyses we excluded all blocks during which the airplane mode was activated. Additionally, for these analyses we had to exclude eight participants for which the Button Box did not accurately record notification sounds. We then replaced the phone value variable with these two variables and once more compared five models. The weights of these model comparisons can be found in Tables 2 and 3, respectively.

**Table 2:** Akaike weights based on Widely Applicable Information Criterion (WAIC) scores of the five task value/# of notifications models predicting the switch from the 2-back task to the smartphone

Model	WAIC	Weight
1 Intercept-only	3,622.7	0.0000
2 Task value	3,607.5	0.0000
3 # of notifications	3,565.4	0.0002
4 Task value + # of notifications	3,548.6	0.8295
5 Task value * # of notifications	3,551.8	0.1703

**Table 3:** Akaike weights based on Widely Applicable Information Criterion (WAIC) scores of the five task value/recent notification models predicting the switch from the 2-back task to the smartphone

Model	WAIC	Weight
1 Intercept-only	3,622.7	0.0000
2 Task value	3,607.5	0.0000
3 Recent notification	3,594.3	0.0027
4 Task value + recent notification	3,582.6	0.9081
5 Task value * recent notification	3,587.3	0.0892

**Fig. 2:**
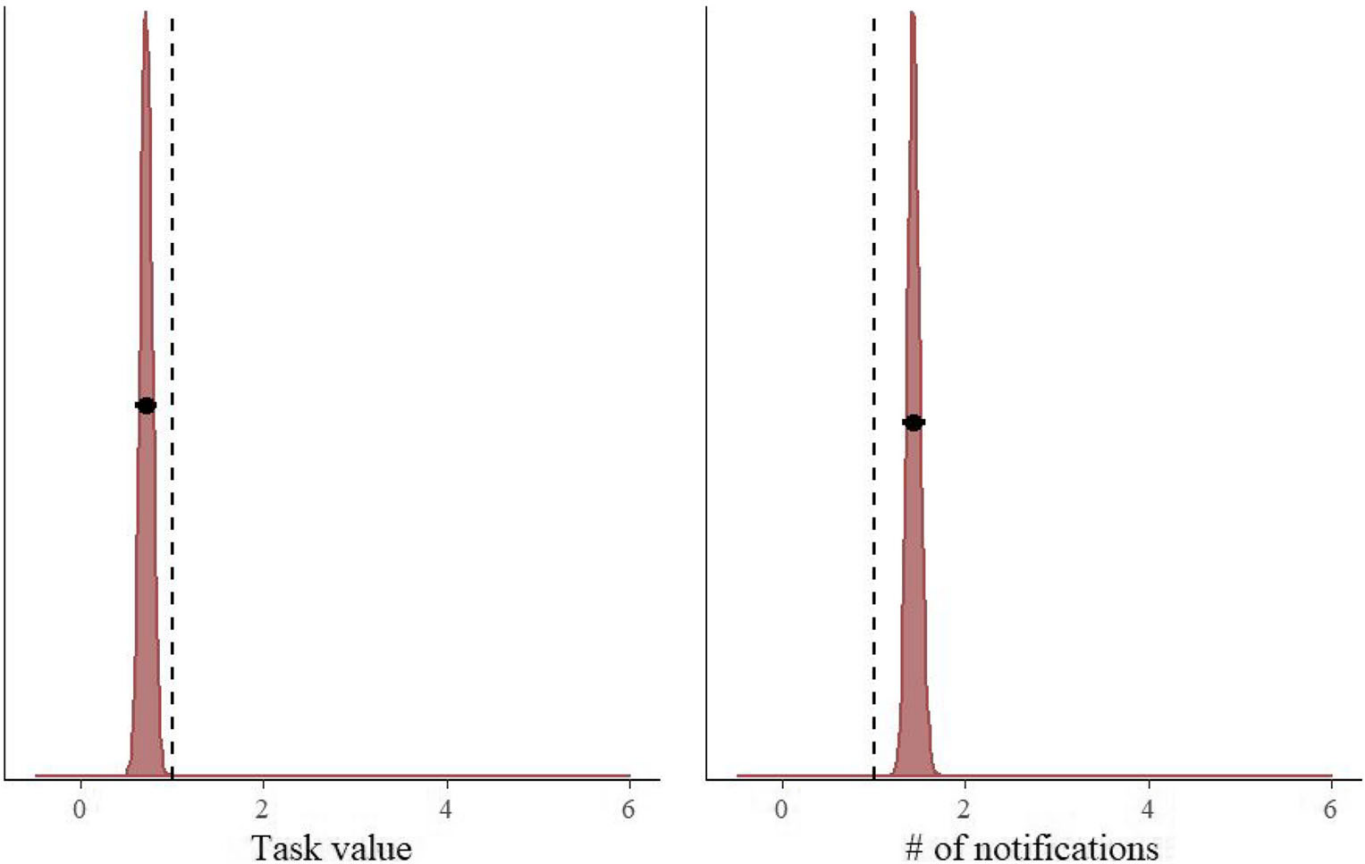
Exponentiated posterior distributions of the parameters (reflecting the odds ratios) for the task value and # of notifications model predicting the switch from the 2-back task to the smartphone. The circles and the lines represent the mean of the posterior and the 95% Bayesian credible interval, respectively

Table 2 shows that the combination of the task value manipulation and the current number of notifications predicted the switch better than the task value alone. This model clearly outperformed the task value-only model. Additionally, this model’s weight was almost five times as large as the next-best model, which included the interaction between the two predictors. The posterior distributions of the “winning” model can be found in Fig. 2. The model estimates that participants were 1.40 times more likely to switch from the 2-back task to the smartphone if the task value is low (95% CI = [1.18, 1.68]), and participants are 1.43 times more likely to switch with each additional notification (95% CI = [1.31, 1.57]).

**Fig. 3:**
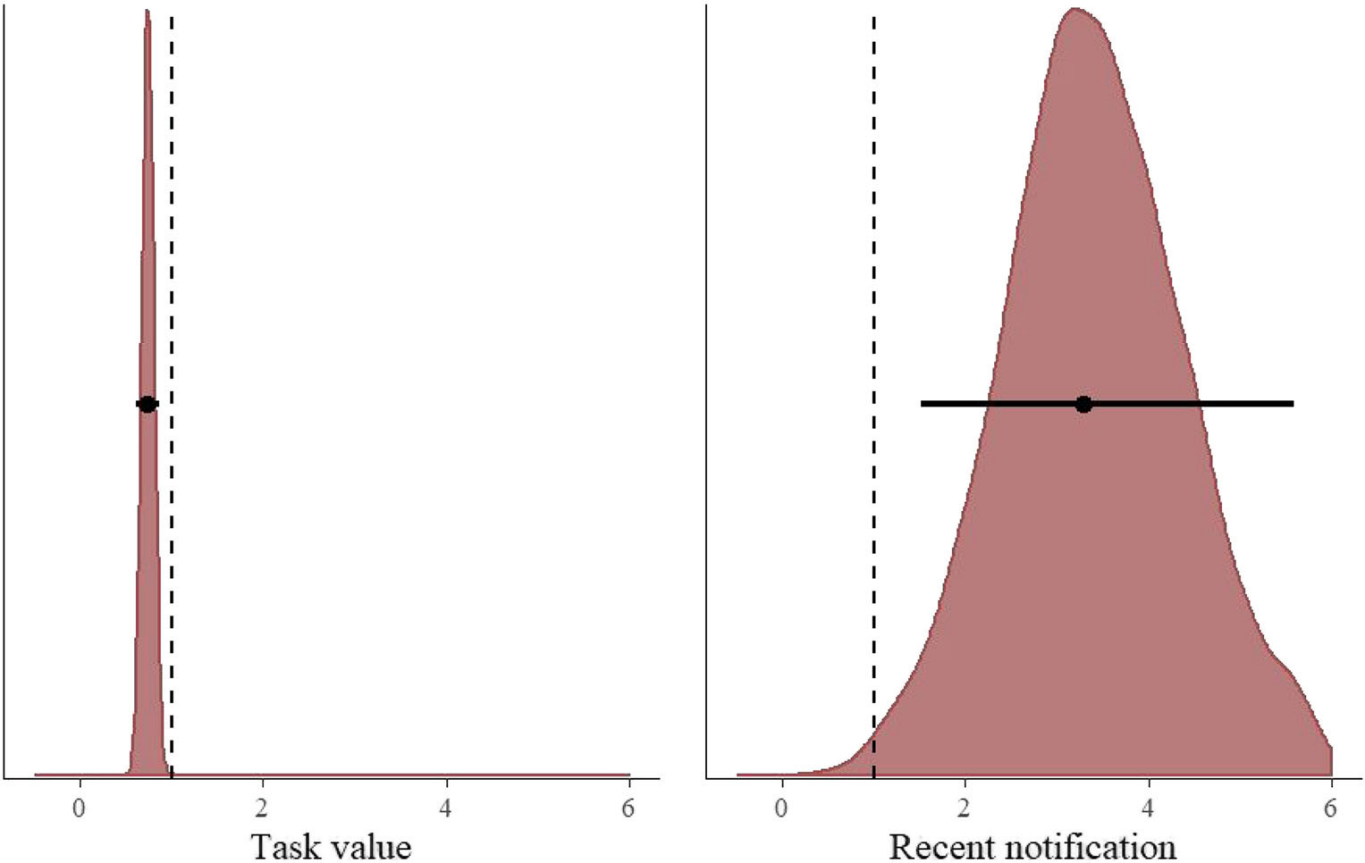
Exponentiated posterior distributions of the parameters (reflecting the odds ratios) for the task value and recent notification model predicting the switch from the 2-back task to the smartphone. The circles and the lines represent the mean of the posterior and the 95% Bayesian credible interval, respectively

Table 3 shows that the model including the task value manipulation and whether the participant received a notification in the past ten trials clearly outperformed the model including only the task value. The weight of this model was more than ten times larger than that of the next-best model. Thus, it appears that participants use both the current task value and recently incoming notifications to decide when to switch from a demanding labor task to the smartphone. The posterior distributions of this model can be found in Fig. 3. The model estimates that participants are 1.35 times as likely to switch to the smartphone when the current task value is low (95% CI = [1.15, 1.62]), and 3.29 times as likely if they received a notification in the past ten 2-back trials (95% CI = [1.52, 5.58]).

### Non-preregistered analyses: Individual differences

Finally, we tested whether individual differences in participants’ baseline valuation of mental labor (i.e., need for cognition) and of interacting with one’s own smartphone (i.e., need for smartphone use) further improved the winning models’ ability to predict labor-to-leisure switches. To do so, we compared the respective three winning models with models that additionally included need for cognition and need for smartphone use. We also included the relevant 2-way interactions (plausibly, need for cognition may moderate the effect of task value; also, need for smartphone use may moderate the effect of incoming notifications). The results of these comparisons were not conclusive and are reported in Table 4. We conclude that there is no clear evidence that individual differences in need for cognition and need for smartphone use improve the respective model’s ability to predict the switch from labor to leisure.

**Table 4:** Akaike weights based on Widely Applicable Information Criterion (WAIC) scores comparing the three winning models with models including the need for cognition and need for smartphone use predicting the switch from the 2-back task to the smartphone

Model	WAIC	Weight
1 Task value		0.5847
2 Task value * need for cognition		0.4153
1 Task value + # of notifications		0.7719
2 Task value * need for cognition + # of notifications * need for smartphone use		0.2281
1 Task value + recent notification		0.7461
2 Task value * need for cognition + recent notification * need for smartphone use		0.2539

## Discussion

With this experiment, we aimed to understand how people decide to switch from a productive-but-demanding task (labor) to interacting with their own smartphone (leisure) in an ecologically valid setting. Our study produced three main findings. First, the current value of the smartphone did not improve our ability to predict the switch from labor to leisure beyond the current value of the task. Second, exploratory analyses showed that people react especially strongly to incoming notifications, which seem to drive the decision when to switch from the task to the smartphone. Third, we found no evidence that taking into account individual differences improved our ability to predict the switch from the task to the smartphone. We now turn to a more detailed discussion of these findings.

Recent theoretical accounts converge on the idea that the decision between labor and leisure depends on the current value of labor *relative* to the current value of leisure (Inzlicht et al., 2014; Kurzban et al., 2013; Shenhav et al., 2017). This idea is relevant to smartphone use; indeed, many people – especially young people – rate the social applications of the smartphone as highly rewarding (Johannes, Dora, & Rusz, 2019). Nevertheless, the availability of these features did not improve our model’s ability to predict the switches from labor to the smartphone when the current value of the labor task was already taken into account. This implies that when people are highly motivated by the current rewards associated with task performance, they are motivated to continue to engage with their task irrelevant of other factors, such as the value of competing activities or stimuli (Rusz, Bijleveld, & Kompier, 2018). Thus, from our preregistered analyses, we conclude that, in practice, high motivation for the current task seems more urgent than removing high-value leisure alternatives from the environment, if one wishes to nudge people to continue to engage in labor.

However, our exploratory analyses suggest that, in addition to considering the current value of the task, people exhibit a strong stimulus-driven response to incoming notifications. This is consistent with the idea that value-related stimuli have the potential to distract people from their work (Anderson, Laurent, & Yantis, 2011; Rusz, Le Pelley, Kompier, Mait, & Bijleveld, 2020). Intriguingly, our findings suggest that the effect of notifications could be substantially stronger than that of task motivation – especially when one considers the recency of these notifications. Together, these results could indicate that the baseline value of the smartphone is not that high, whether all of its features are available or not, but that the value sharply increases with incoming notifications. Previous work suggests that the value of real-world leisure alternatives likely varies considerably from moment to moment (Dora et al., 2019; Kool & Botvinick, 2014; Kurzban et al., 2013); our findings suggest that smartphones substantially contribute to this variability in practice.

Our study has two important practical implications. First, it highlights the importance of high task motivation for people to persist in engaging in their labor tasks. Even while engaging with a highly demanding cognitive task, this motivation mostly prevented participants from taking breaks with their smartphone – at least when participants did not receive notifications. Thus, rather than worrying about the availability of leisure alternatives in the environment, people should attempt to keep the value of their labor task high. For instance, high task motivation can be achieved by setting specific, challenging, and meaningful goals (Locke & Latham, 2006; Locke & Latham, 2019).

Second, in line with previous research on the effect of smartphone notifications on cognition (Kushlev, Proulx, & Dunn, 2016; Shelton, Elliott, Eaves, & Exner, 2009; Stothart, Mitchum, & Yehnert, 2015), our results highlight the disruptive potential of such incoming notifications. Whereas our participants managed to engage with their task for the majority of the time, receiving a notification within the last 20 s increased the odds of a switch from labor to leisure by more than three. While some evidence indicates that short smartphone interactions may help people to recover from fatigue (Dora et al., 2019), our results clearly suggest that people should temporarily turn off the notifications on their smartphone during periods of productivity, such as office hours and while sitting in the library preparing for an important test. This strategy may also help to prevent procrastination. Indeed, several studies (Meier, Reinecke, & Meltzer, 2016; Reinecke & Hofmann, 2016; Schnauber-Stockmann, Meier, & Reinecke, 2018) showed that procrastination via the smartphone is common, especially among students and young adults. Our results indicate that turning off notifications may curb this type of procrastination.

Related to our recommendation to switch off notifications during focused work, it is important to note that some research indicates that smartphone users themselves do not recognize the potential problems associated with smartphone distractions (Berry & Westfall, 2015). Whereas previous accounts have called for the implementation of policies removing smartphones altogether from classrooms and workplaces (and the difficulties in getting people to accept such policies; Berry & Westfall, 2015; Campbell, 2006; Gill, Kamath, & Gill, 2012), our data suggest that merely turning off notifications (paired with high task motivation) may go a long way in keeping productivity high when it is most important.

It is worthwhile considering how our theoretical framework, which distinguishes between labor and leisure, relates to other models of motivation. First, the distinction between labor and leisure is reminiscent of the distinction between *extrinsic motivation* and *intrinsic motivation* (e.g., Deci & Ryan, 2002). Specifically, in our framework, we assume that labor is an extrinsically motivated behavior, whereas leisure may often be an intrinsically motivated behavior (Kool & Botvinick, 2014). We should note, however, that real-life labor tasks are not always exclusively motivated by external rewards. For example, even though employees get paid by their employers (an external reward), they may well find their work meaningful and important, and thus derive pleasure from the work itself (an internal reward). So, the labor–leisure dimension may often map onto the extrinsic–intrinsic dimension, but this mapping is imperfect. Second, the distinction between labor and leisure is reminiscent of the distinction between *wants* (i.e., choice options that have instantaneous utility) and *shoulds* (i.e., choice options that have utility in the long term; Bitterly, Mislavsky, Dai, & Milkman, 2015). Although the *want–should conflict* model emphasizes the temporal dimension of goal pursuit more than the labor–leisure model does, it is reasonable to assume that labor is often a *should* and that leisure is often a *want* (Inzlicht et al., 2014). Thus, these three research areas (i.e., the labor–leisure model, self-determination theory, and the literature on want–should conflicts) are related, and there is potential for cross-fertilization.

One limitation of our study is that our operationalization of labor (the 2-back task) was not as ecologically valid as our operationalization of leisure (interacting with one’s own smartphone). We made this choice because we wanted to keep some control over the demands placed on participants during labor. Now we have studied how people decide to switch from labor to leisure when labor is highly structure and not self-paced, future research could investigate whether this decision is made differently when people work on more natural labor tasks. One way this could be done is to invite students to study in the lab for an upcoming exam (with their smartphone on the table) and observe them for a fixed period of time. Alternatively, one could make use of experience-sampling designs to study the temporal dynamics of labor/leisure decisions (Dora et al., 2020; Hofmann, Baumeister, Förster, & Vohs, 2012).

One previous study (Yeykelis, Cummings, & Reeves, 2014) that investigated task switching during computer use in a natural setting found that participants switched between tasks much more frequently than participants did in our experiment. One possible explanation might be so-called demand effects where participants change their behavior in order to display perceived appropriate behavior during the experiment (Zizzo, 2010). We tried to minimize these demand effects by instructing participants that they were free to distribute their time between the two tasks in whichever way they wanted to. Alternatively, the differences may stem from the fact that in our study a switch was operationalized as a choice between two very different activities and devices, while in Yeykelis et al. (2014) switches were observed between tabs on a computer. Future research should investigate these possibilities.

Furthermore, it is important to note that the ecological validity of our experiment may have been further limited by the fact that we explicitly instructed participants to continuously choose between the 2-back task and their own smartphone. In real life, the decision to pick up the smartphone is often formed outside of conscious awareness (Bayer & LaRose, 2018). In order to test whether our results reflect this more natural decision-making process, future research should use experience-sampling designs involving log data from the participants’ smartphones.

A second limitation of our study was that the results likely were dependent on the specific manipulations of labor and leisure value chosen. For example, had we offered participants ten times the amount of money during high labor-value blocks compared to low labor-value blocks, the labor value manipulation may well have had an even stronger effect on participants’ decision-making. We should note, however, that these potential alternative results would not challenge the conclusions we draw from the present research. Still, an important next step for future research is to investigate how labor versus leisure decision-making is affected when labor and leisure values fluctuate along a continuum. On a related note, the results might have been affected by the fact that participants were well aware when the current value of the task was high versus low. One open question is whether our results would replicate in a setting where this value is hidden, for example when the labor value is manipulated between participants. To answer this question while ensuring that the value of labor relative to leisure is high (in the high-value treatment) versus low (in the low-value treatment), future research could use a discounting paradigm to establish a point of indifference for each participant (e.g., Westbrook et al., 2013), and then increase versus decrease the payment offered for the labor task by a fixed amount.

A third limitation of our study was that we did not offer participants additional leisure alternatives. Most of the time, real life does not only offer people the smartphone as an alternative to their labor tasks, and people rarely put their smartphone in airplane mode. Hence, future work may expand on the current study by studying smartphone use as one of a range of leisure alternatives to some labor task.

## Conclusion

The present study enriches the scientific literature on labor/leisure tradeoffs by examining how people switch from labor to leisure in a setting high in ecological validity. With this situated approach (Hutchins, 1995), we showed that the combination of task motivation and a temporary boost in leisure value through incoming notifications best explained the decision to disengage from a productive-but-demanding task to switch to the smartphone. In future research, it may well be useful to study additional labor/leisure decisions in their naturally occurring environment.

## Footnotes

1 We ensured that participants manually turn off the Wi-Fi along with turning on the airplane mode on those devices that do not disconnect from Wi-Fi during airplane mode.

## References

[CR1] Algermissen J, Bijleveld E, Jostmann N, Holland R (2019). Pupil diameter transiently increases in self-chosen switches between cognitive labor and leisure in either direction. Cognitive, Affective, & Behavioral Neuroscience.

[CR2] Anderson B, Laurent P, Yantis S (2011). Value-driven attentional capture. Proceedings of the National Academy of Sciences.

[CR3] Barr D, Levy R, Scheepers C, Tily H (2013). Random effects structure for confirmatory hypothesis testing: Keep it maximal. Journal of Memory and Language.

[CR4] Bayer, J., & LaRose, R. (2018). Technology habits: Progress, problems and prospects. In *The psychology of habit* (pp. 111 – 130). Springer.

[CR5] Berry M, Westfall A (2015). Dial D for Distraction: The Making and Breaking of Cell Phone Policies in the College Classroom. College Teaching.

[CR6] Bitterly, T. B., Mislavsky, R., Dai, H., & Milkman, K. L. (2015). Want-should conflict: A synthesis of past research. In W. Hoffman & L. Nordgren (Eds.), *The psychology of desire* (pp. 244–266). Guilford.

[CR7] Bürkner P (2017). brms: An R package for Bayesian multilevel models using Stan. Journal of Statistical Software.

[CR8] Cacioppo J, Petty R, Feng Kao C (1984). The efficient assessment of need for cognition. Journal of personality assessment.

[CR9] Campbell S (2006). Perceptions of mobile phones in college classrooms: Ringing, cheating, and classroom policies. Communication education.

[CR10] Clancey, W. (1997). *Situated cognition: On human knowledge and computer representations*. Academic Press.

[CR11] Deci, E., & Ryan, R. (2002). *Handbook of self-determination research*, University of Rochester Press.

[CR12] Dora, J., van Hooff, M., Geurts, S., Kompier, M., & Bijleveld, E. (2019). The effect of opportunity costs on mental fatigue in labor/leisure tradeoffs. 10.31234/osf.io/3765s 10.1037/xge0001095 34472958

[CR13] Dora, J., van Hooff, M., Geurts, S., Kompier, M., & Bijleveld, E. (2020). Fatigue, boredom, and objectively-measured smartphone use at work. 10.31234/osf.io/uy8rs 10.1098/rsos.201915 PMC8261226 34295513

[CR14] Gill P, Kamath A, Gill T (2012). Distraction: an assessment of smartphone usage in health care work settings. Risk management and healthcare policy.

[CR15] Goldman S, Scardamalia M (2013). Managing, Understanding, Applying, and Creating Knowledge in the Information Age: Next-Generation Challenged and Opportunities. Cognition and Instruction.

[CR16] Hockey, R. (2011). A motivational control theory of fatigue. In P. Ackerman (Ed.), *Cognitive fatigue: multidisciplinary perspectives on current research and future applications* (pp. 167 – 188). American Psychological Association.

[CR17] Hofmann W, Baumeister R, Förster G, Vohs K (2012). Everyday temptations: an experience sampling study of desire, conflict, and self-control. Journal of personality and social psychology.

[CR18] Hutchins, E. (1995). *Cognition in the wild*. The MIT Press.

[CR19] Inzlicht M, Schmeichel B, Macrae C (2014). Why self-control seems (but may not be) limited. Trends in cognitive sciences.

[CR20] Johannes N, Dora J, Rusz D (2019). Social Smartphone Apps Do Not Capture Attention Despite Their Perceived High Reward Value. Collabra: Psychology.

[CR21] Kool W, Botvinick M (2014). A labor/leisure tradeoff in cognitive control. Journal of Experimental Psychology: General.

[CR22] Kurzban R, Duckworth A, Kable J, Myers J (2013). An opportunity cost model of subjective effort and task performance. Behavioral and brain sciences.

[CR23] Kushlev, K., Proulx, J., & Dunn, E. (2016). Silence your phones: Smartphone notifications increase inattention and hyperactivity symptoms. In *Proceedings of the 2017 CHI conference on human factors in computing systems* (pp. 1011 – 1020).

[CR24] Lin, H., Werner, K., & Inzlicht, M. (2020). Promises and Perils of Experimentation: Big-I Triangulation Offers Solutions. 10.31234/osf.io/hwubj

[CR25] Lo S, Andrews S (2015). To transform or not to transform: using generalized linear mixed models to analyze reaction time data. Frontiers in Psychology.

[CR26] Locke E, Latham G (2006). New directions in goal-setting theory. Current directions in psychological science.

[CR27] Locke E, Latham G (2019). The development of goal setting theory: A half century retrospective. Motivation Science.

[CR28] Markman A, Dietrich E (2000). Something old, something new: Extending the classical view of representation. Trends in Cognitive Sciences.

[CR29] Meier A, Reinecke L, Meltzer C (2016). “Facebocrastination”? Predictors of using Facebook for procrastination and its effects on students’ well-being. Computers in Human Behavior.

[CR30] Oulasvirta A, Rattenbury T, Ma L, Raita E (2012). Habits make smartphone use more pervasive. Personal and Ubiquitous Computing.

[CR31] Peirce J (2007). PsychoPy – psychophysics software in Python. Journal of neuroscience methods.

[CR32] R Core Team (2020). R: A language and environment for statistical computing. *R Foundation for Statistical Computing*. Retrieved from https://www.r-project.org

[CR33] Reinecke, L., & Hofmann, W. (2016). Slacking Off or Winding Down? An Experience Sampling Study on the Drivers and Consequences of Media Use for Recovery Versus Procrastination. *Human Communication Research, 42*, 441 – 461. 10.1111/hcre.12082

[CR34] Rom, S., Katzir, M., Diel, K., & Hofmann, W. (2019). On trading off labor and leisure: A process model of perceived autonomy and opportunity costs. *Motivation Science,*10.1037/mot0000148

[CR35] Rosen L, Whaling K, Carrier L, Cheever N, Rokkum J (2013). The media and technology usage and attitudes scale: An empirical investigation. Computers in human behavior.

[CR36] Rusz D, Bijleveld E, Kompier M (2018). Reward-associated distractors can harm cognitive performance. Plos One.

[CR37] Rusz, D., Le Pelley, M., Kompier, M., Mait, L., & Bijleveld, E. (2020). Reward-driven distraction: A meta-analysis. 10.31234/osf.io/82csm 10.1037/bul0000296 32686948

[CR38] Schnauber-Stockmann A, Meier A, Reinecke L (2018). Procrastination out of habit? The role of impulsive versus reflective media selection in procrastinatory media use. Media Psychology.

[CR39] Shelton J, Elliott E, Eaves S, Exner A (2009). The distracting effects of a ringing cell phone: An investigation of the laboratory and the classroom setting. Journal of environmental psychology.

[CR40] Shenhav A, Musslick S, Lieder F, Kool W, Griffiths T, Cohen J (2017). Toward a rational and mechanistic account of mental effort. Annual review of neuroscience.

[CR41] Stothart C, Mitchum A, Yehnert C (2015). The attentional cost of receiving a cell phone notification. Journal of Experimental Psychology: Human Perception and Performance.

[CR42] Vehtari A, Gelman A, Gabry J (2017). Practical Bayesian model evaluation using leave-one-out cross-validation and WAIC. Statistics and computing.

[CR43] Watanabe S (2010). Asymptotic equivalence of Bayes cross validation and widely applicable information criterion in singular learning theory. Journal of Machine Learning Research.

[CR44] Westbrook A, Kester D, Braver T (2013). What is the subjective cost of cognitive effort? Load, trait, and aging effects revealed by economic preference. Plos One.

[CR45] Yeykelis L, Cummings J, Reeves B (2014). Multitasking on a single device: Arousal and the frequency, anticipation, and prediction of switching between media content on a computer. Journal of Communication.

[CR46] Zizzo D (2010). Experimenter demand effects in economic experiments. Experimental Economics.

